# Acute myocarditis after the third dose of COVID‐19 mRNA‐1273 vaccine

**DOI:** 10.1002/jgf2.604

**Published:** 2023-01-18

**Authors:** Takayuki Yamada

**Affiliations:** ^1^ Asunaro Clinic Takasaki Japan

**Keywords:** cardiovascular medicine, family medicine

## Abstract

Myocarditis caused by the mRNA‐1273 coronavirus disease 2019 vaccine must be considered for patients complaining of acute constant general fatigue postvaccination.

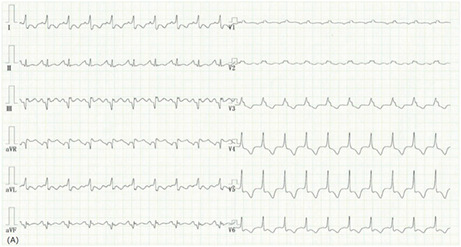

A 43‐year‐old previously healthy man presented with acute diarrhea and constant general fatigue for 11 days. In addition, he complained of a cough and arthralgia that persisted for 2 days. Twenty‐two days earlier, he received his third dose of the mRNA‐1273 (Moderna) coronavirus disease 2019 (COVID‐19) vaccine. Prior to this illness, he had no contact with any individuals with fever, including those positive for severe acute respiratory syndrome coronavirus 2. After the first two vaccine doses, he developed a moderate fever that lasted for 1 day on both occasions. Upon arrival at our clinic, he appeared unwell, and his vital signs were as follows: blood pressure of 122/90 mmHg, heart rate of 125 beats/min, body temperature of 36.3°C, respiratory rate of 26 breaths/minute, and transcutaneous oxygen saturation of 93% in room air. His COVID‐19 antigen test result was negative. An electrocardiogram (Figure 1) revealed inverted T waves in precordial leads V3–V6 and aVL. Echocardiography revealed diffuse left ventricular dysfunction indicating acute myocarditis with heart failure. Consequently, he was immediately admitted to a hospital. His laboratory tests revealed an elevated C‐reactive protein level of 14.4 mg/dl, aspartate aminotransferase of 511 U/L, alanine aminotransferase of 575 U/L, and NT‐proB‐type natriuretic peptide of 3433 pg/ml. Diuretic administration and bed rest improved his medical condition. Moreover, he was discharged from the hospital after 9 days. The incidence of myocarditis subsequent to the second dose of the vaccine is higher than after the first dose. The incidence of myocarditis following the third dose of vaccine did not appear significantly higher than that observed after the second dose of vaccine.^1^ Although COVID‐19 vaccine‐associated myocarditis is rare,^2^ it must be considered in patients complaining of constant general fatigue postvaccination.

**FIGURE 1:**
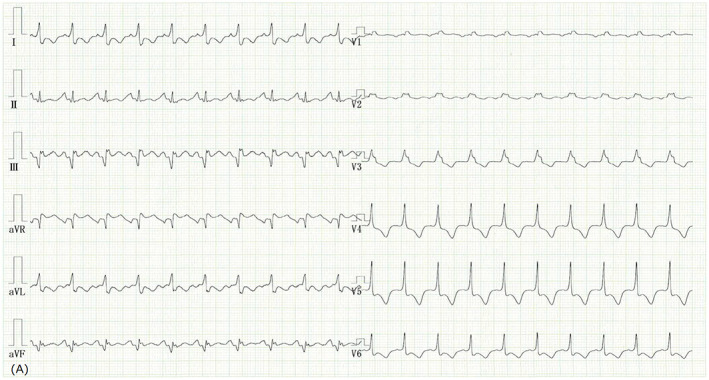
An electrocardiogram revealing inverted T waves in precordial leads V3–V6 and aVL.

## CONFLICT OF INTEREST

The author declare no conflict of interest.

## ETHICS APPROVAL STATEMENT

Ethical approval was not required for this study.

## PATIENT CONSENT STATEMENT

Written informed consent was obtained from the patient to publish this report as per the journal's patient consent policy.
